# Context-dependent pro- and anti-resection roles of ZKSCAN3 in the regulation of fork processing during replication stress

**DOI:** 10.1016/j.jbc.2022.102215

**Published:** 2022-06-30

**Authors:** Zheng Yang, Delphine Sangotokun Lemacon, Shan Li, Abigael Cheruiyot, Lingzhen Kong, Ke Tan, Chen Cheng, Ecenur Turkay, Dalin He, Zhongsheng You

**Affiliations:** 1 Department of Urology, First Affiliated Hospital of Xi’an Jiaotong University, Xi’an, Shaanxi, China; 2 Department of Cell Biology and Physiology, Washington University School of Medicine, St Louis, Missouri, USA

**Keywords:** replication stress, fork resection, ZKSCAN3, BRCA1, chemosensitivity, CldU, chlorodeoxyuridine, DSB, double-strand DNA break, HR, homologous recombination, HU, hydroxyurea, IdU, iododeoxyuridine, PARPi, PARP inhibitors, PBS, phosphate-buffered saline, PBST, Triton X-100 in PBS, ZNF, zinc finger

## Abstract

Uncontrolled resection of replication forks under stress can cause genomic instability and influence cancer formation. Extensive fork resection has also been implicated in the chemosensitivity of “BReast CAncer gene” BRCA-deficient cancers. However, how fork resection is controlled in different genetic contexts and how it affects chromosomal stability and cell survival remains incompletely understood. Here, we report a novel function of the transcription repressor ZKSCAN3 in fork protection and chromosomal stability maintenance under replication stress. We show disruption of ZKSCAN3 function causes excessive resection of replication forks by the exonuclease Exo1 and homologous DNA recombination/repair protein Mre11 following fork reversal. Interestingly, in BRCA1-deficient cells, we found ZKSCAN3 actually promotes fork resection upon replication stress. We demonstrate these anti- and pro-resection roles of ZKSCAN3, consisting of a SCAN box, Kruppel-associated box, and zinc finger domain, are mediated by its SCAN box domain and do not require the Kruppel-associated box or zinc finger domains, suggesting that the transcriptional function of ZKSCAN3 is not involved. Furthermore, despite the severe impact on fork structure and chromosomal stability, depletion of ZKSCAN3 did not affect the short-term survival of BRCA1-proficient or BRCA1-deficient cells after treatment with cancer drugs hydroxyurea, PARPi, or cisplatin. Our findings reveal a unique relationship between ZKSCAN3 and BRCA1 in fork protection and add to our understanding of the relationships between replication fork protection, chromosomal instability, and chemosensitivity.

Complete, faithful replication of DNA is key to genome maintenance and cell proliferation (1). However, the DNA replication process, which is carried out by tens of thousands of replication forks, is frequently perturbed by factors of both exogenous and endogenous origins (2, 3). To cope with this challenge, cells have evolved a multitude of mechanisms to protect the fork structure upon replication stress and promote replication restart when the stress condition is resolved (4). A number of chromatin-associated factors (*e.g.*, BRCA1, BRCA2, Rad51, BOD1L, FANC proteins) have been identified to be important for replication fork protection, which is essential for the prevention of mutations and genomic instability (5). The ATR/Chk1-dependent checkpoint pathway and a newly identified Ca^2+^-dependent signaling pathway have also been shown to promote the protection of stalled replication forks (6, 7). Despite these important advances, the molecular mechanisms and regulation of fork protection and its role in cell survival in the presence of replication stress remain incompletely understood.

Defects in fork protection are considered a major underlying mechanism of genomic instability caused by mutations in fork protection factors (5). The importance of fork protection in cancer avoidance and treatment is exemplified by the functions of the tumor suppressor BRCA1 (8, 9). BRCA1 protects genome stability through its role in both DNA repair by homologous recombination (HR) and replication fork protection (8, 9, 10). BRCA1 promotes HR, in part, by promoting resection at double-strand DNA break (DSB) ends (11, 12, 13, 14). Paradoxically, at stalled replication forks, BRCA1 acts to prevent excessive fork resection by nucleases including Mre11 and Exo1 that are also involved in DSB resection (15). Indeed, BRCA1-deficient cancer cells exhibit excessive fork DNA resection when challenged with replication stressors such as hydroxyurea (HU) (8, 15). This aberrant fork resection is believed to occur after fork reversal, leading to degradation of nascent DNA and generation of extensive ssDNA behind the replication forks, which eventually gives rise to chromosomal instability (16, 17). The defects in HR and fork protection caused by BRCA1-deficiency confer cellular hypersensitivity to PARP inhibitors (PARPi) (18, 19). At least four PARPi (Olaparib, Rucaparib, Talazoparib, Niraparib), which are synthetic lethal with BRCA1-deficiency, have been approved by FDA for treating various cancers. However, cancer resistance to these drugs develops almost invariably following treatment. It was suggested that restoration of fork protection is a major mechanism underlying the acquired chemoresistance, although later studies suggest that this relationship is dependent on specific genetic contexts (20, 21). Thus, further elucidation of the regulation of fork protection in both BRCA1-proficient cells and BRCA1-deficient cells is important for the understanding of the mechanisms of genome maintenance and the development of novel approaches to overcome cancer resistance to PARPi.

In an effort to identify new players and regulators in the replication fork protection process, we explored the potential involvement of a group of ZKSCAN proteins. ZKSCAN proteins belong to the zinc finger (ZNF)-containing protein family, which consists of the largest group of DNA-binding proteins and are encoded by 5% of human protein-coding genes (22). In the ZNF superfamily, 718 members contain the C2H2-type ZNF domain and most of them are transcription factors (23). Several of these factors have been implicated in genome maintenance (*e.g.*, TZAP, APTX, RNF138, ZATT) (24). ZKSCAN is a unique subfamily of ZNF-containing proteins that contain both KRAB domain and SCAN box in addition to C2H2 ZNF domains. Twenty-five ZKSCAN genes have been identified in human based on their predicted protein sequences, although most of them have not been functionally characterized (25). These ZKSCAN proteins likely act as transcriptional repressors due to the presence of the KRAB domain (26). The SCAN box is believed to mediate protein dimerization (homodimerization or heterodimerization), although its exact role in these proteins remains to be defined (27, 28, 29, 30). ZKSCAN3 is one of the few ZKSCAN proteins that have been studied previously. ZKSCAN3 acts to suppress autophagy through its role in inhibiting the transcription of multiple genes that are required for autophagy and lysosome biogenesis (31). A more recent study suggests that ZKSCAN3 also facilitates heterochromatin maintenance to suppress senescence, and that this function is independent of its transcriptional regulation of autophagy (32). Aberrant expression of ZKSCAN3 has been shown to be associated with enhanced cell proliferation and cancer metastasis, although the exact role of ZKSCAN3 dysregulation in tumorigenesis remains to be defined (33, 34, 35, 36, 37, 38, 39, 40).

By carrying out a siRNA screen on the ZKSCAN protein family using a fork resection assay, we identified ZKSCAN3 as a new factor required for fork protection in BRCA1-proficient cells in the presence of replication stress. Surprisingly, in BRCA1-deficient cells, ZKSCAN3 promotes, instead of inhibiting, fork resection upon replication stress. Remarkably, the SCAN box alone is both necessary and sufficient for the context-dependent role of ZKSCAN3 in the regulation of fork resection. Despite the roles of ZKSCAN3 in fork resection and chromosomal stability in both BRCA1-proficient and BRCA1-deficient cells, its depletion did not obviously affect the sensitivity of those cells to HU, cisplatin, or Olaparib.

## Results

### ZKSCAN3 is a novel fork protection factor required for chromosome stability under replication stress

**Figure 1:**
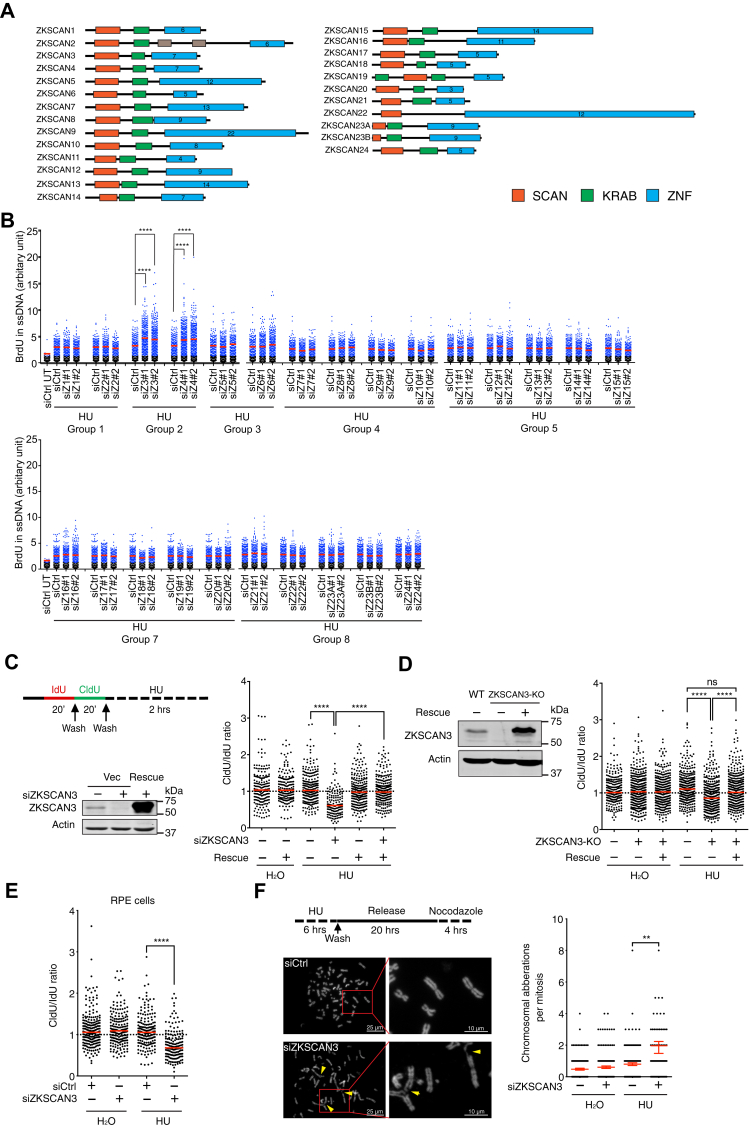
**ZKSCAN3 promotes fork resection and chromosomal stability under replication stress.***A*, schematic diagram of the ZKSCAN proteins family. Numbers shown in *blue box* denote the number of the Zinc fingers in each protein. *B*, a siRNA screen of ZKSCAN proteins as potential fork protection factors. Fork resection was measured in HeLa cells after HU treatment (2 mM, 5 h) using a nondenaturing BrdU-staining assay. Cells with BrdU signal higher than the majority (98%) of untreated control cells (*black dots*) were taken as BrdU-positive (*blue dots*). *Red bars* represent the mean BrdU intensity of BrdU-positive cells. HU-treated, control siRNA (siCtrl)-transfected HeLa cell samples were shared in each experiment group. Thousand cells were analyzed for each sample. n = 2, ∗∗∗∗*p* ≤ 0.0001 (unpaired *t* test). *C*, effects of ZKSCAN3 depletion on fork resection after replication stress. *Left upper panel*: Experimental scheme (see Experimental procedures). *Left lower panel*: siRNA-mediated knockdown of ZKSCAN3 in HeLa cells and expression of siRNA-resistant ZKSCAN3 in knockdown cells. *Right panel*: Ratio of CldU/IdU *track lengths* for the samples depicted in the left lower panel. *Red bars* represent the median. At least, 200 tracks were measured for each sample. n = 3, ∗∗∗∗*p* ≤ 0.0001 (one-way ANOVA). *D*, effects of ZKSCAN3 KO on fork resection after replication stress. *Left panel*: Expression of FLAG-tagged ZKSCAN3 in ZKSCAN3-KO cells. *Right panel*: Ratio of CldU/IdU *track lengths* for the samples depicted in the *left lower panel*. *Red bars* represent the median. At least 200 tracks were scored for each sample. n = 3, ∗∗∗∗*p* ≤ 0.0001, ns, not significant (one-way ANOVA). *E*, effects of ZKSCAN3 knockdown on fork resection in RPE cells after HU treatment (4 mM, 2 h). *Red bars* represent the median. At least 200 tracks were scored for each sample. n = 3, ∗∗∗∗*p* ≤ 0.0001 (unpaired *t* test). *F*, effects of ZKSCAN3 depletion on chromosomal integrity under replication stress. HeLa cells transfected with control- or ZKSCAN3-siRNAs were treated with HU (4 mM, 6 h) before the metaphase chromosomal spreading assay. *Left panel*: Representative images of metaphase chromosome spreads in control- or ZKSCAN3-depleted cells treated with HU. Chromosomal aberrations are marked by *arrows*. *Right panel*: Quantified results of the samples depicted in the *left panel*. One hundred fifty metaphases were examined for each sample. n = 3, ∗∗*p* ≤ 0.01 (unpaired *t* test). CldU, chlorodeoxyuridine; HU, hydroxyurea; IdU, iododeoxyuridine.

To explore the potential involvement of the human ZKSCAN family proteins in genome maintenance, we performed a siRNA screen to test their potential roles in preventing abnormal fork resection after replication stress. We used a previously described native BrdU immunofluorescence staining assay, which detects exposed ssDNA in cells, to assess fork resection under replication stress (7). Two different siRNAs were transfected into HeLa cells to silence each of the 25 ZKSCAN genes (Fig. 1*A*). Cells were then incubated with BrdU for 36 h, followed by HU treatment for 5 h to induce replication stress. After this, cells were permeabilized, fixed, and stained with an anti-BrdU antibody. As shown in Figure 1*B*, while siRNAs targeting most of ZKSCANs did not cause an overt fork resection phenotype, siRNAs targeting ZKSCAN3 or ZKSCAN4 resulted in a significant increase of anti-BrdU signal in HU-treated cells, suggesting that these two ZKSCAN proteins are important for fork protection in the presence of replication stress. In this study, we choose to focus on ZKSCAN3, in part because its aberrant expression has been observed in multiple cancers (33, 34, 35, 36, 37, 38, 39, 40).

To further validate the function of ZKSCAN3 in fork protection, we performed a DNA fiber assay that measures the degradation of nascent DNA strands at replication forks as a result of fork resection. HeLa cells were incubated with thymidine analogs iododeoxyuridine (IdU) and chlorodeoxyuridine (CldU) sequentially followed by HU treatment to induce replication stress. Cells were then lysed and genomic DNA was stretched on slides by gravity. IdU and CldU tracks in the DNA fibers were then detected by immunofluorescence and the track lengths were measured. A ratio of CldU/IdU < 1 represents fork resection (41). Consistent with the native BrdU assay results, siRNA-mediated knockdown of ZKSCAN3 caused an elevated level of nascent DNA degradation in HU-treated cells. Importantly, this fork resection phenotype was fully rescued by expression of an siRNA-resistant ZKSCAN3 construct in ZKSCAN3-depleted cells (Fig. 1*C*). To further confirm the requirement of ZKSCAN3 for fork protection, we generated a ZKSCAN3-KO HeLa cell line using the CRISRP/Cas9 method. As shown in Figure 1*D*, ZKSCAN3-KO cells exhibited a higher level of fork resection, than parental cells, after HU treatment. This fork resection phenotype was again rescued by ectopically expressed ZKSCAN3. siRNA-mediated knockdown of ZKSCAN3 in the nontransformed RPE cell line also resulted in excessive fork resection after replication stress (Fig. 1*E*). Together, these results indicate that ZKSCAN3 plays a key role in preventing aberrant fork resection after replication stress.

To further elucidate the significance of the fork protection function of ZKSCAN3, we next performed a chromosome spreading assay to analyze the effects of ZKSCAN3 depletion on chromosome structural integrity. As shown in Figure 1*F*, ZKSCAN3-knockdown cells exhibited an elevated level of chromosomal abnormalities (including both breaks and fusions) after HU treatment, compared to control-knockdown cells. Taken together, these data strongly suggest that ZKSCAN3 is a novel fork protection factor that safeguards chromosomal stability in the presence of replication stress.

### ZKSCAN3 prevents aberrant fork resection by Exo1 and Mre11 after fork reversal

**Figure 2:**
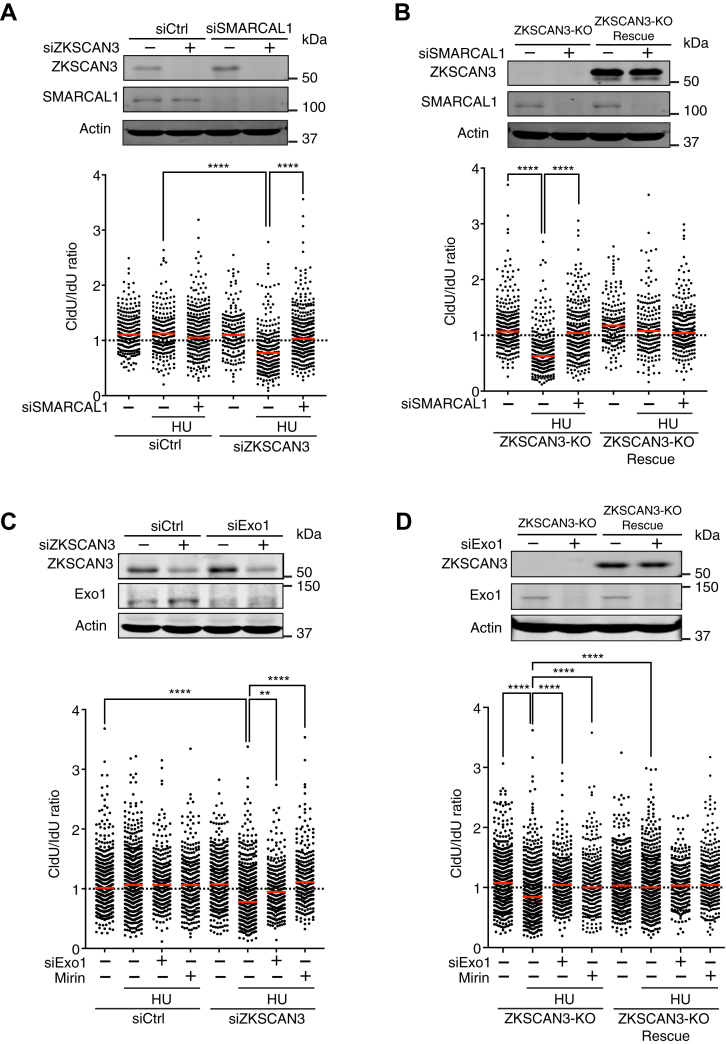
**ZKSCAN3 prevents aberrant fork resection by Exo1 and Mre11 after fork reversal.***A*, effects of SMARCAL1 depletion on fork resection in ZKSCAN3-knockdown HeLa cells after replication stress. *Upper panel*: siRNA-mediated knockdown of SMARCAL1 in ZKSCAN3-depleted cells. *Lower panel*: Results of DNA fiber analysis for measuring fork resection in cells transfected with siRNA targeting ZKSCAN3, SMARCAL1, or both after HU treatment (4 mM, 2 h). *Red bars* represent the median. At least 200 tracks were scored for each sample. n = 3, ∗∗∗∗*p* ≤ 0.0001 (one-way ANOVA). *B*, effects of SMARCAL1 depletion on fork resection in ZKSCAN3-KO HeLa cells after replication stress. *Upper panel*: siRNA-mediated knockdown of SMARCAL1 in ZKSCAN3-KO and reconstituted HeLa cells. *Lower panel*: Fork resection in ZKSCAN3-KO cells or reconstituted cells after HU treatment (4 mM, 2 h). *Red bars* represent the median. At least 200 tracks were scored for each sample. n = 3, ∗∗∗∗*p* ≤ 0.0001 (one-way ANOVA). *C*, effects of Exo1 depletion or Mre11 inhibition on fork resection in ZKSCAN3-knockdown HeLa cells after replication stress. *Upper panel*: siRNA-mediated knockdown of Exo1 in ZKSCAN3-depleted cells. *Lower panel*: Fork resection in ZKSCAN3-knockdown cells treated with Exo1 siRNA or with Mirin (50 mM) after HU treatment (4 mM, 2 h). *Red bars* represent the median. At least 200 tracks were scored for each sample. n = 3, ∗∗∗∗*p* ≤ 0.0001, ∗∗*p* ≤ 0.01 (one-way ANOVA). *D* effects of Exo1 depletion or Mre11 inhibition on fork resection in ZKSCAN3-KO HeLa cells after replication stress. *Upper panel*: siRNA-mediated knockdown of Exo1 in ZKSCAN3-KO or reconstituted cells. *Lower panel*: Fork resection in ZKSCAN3-KO or reconstituted cells treated with Exo1 siRNA or with Mirin (50 mM) after HU treatment (4 mM, 2 h). *Red bars* represent the median. At least 200 tracks were scored for each sample. n = 3, ∗∗∗∗*p* ≤ 0.0001 (one-way ANOVA). HU, hydroxyurea.

Excessive fork resection usually occurs on reversed forks in the presence of replication stress (16). Fork reversal is believed to occur through two separate pathways, with one mediated by FBH1 and the other by SMARCAL1, ZRANB3, and HLTF (42, 43, 44). In the presence of HU, fork reversal requires SMARCAL1 (45). To determine whether fork reversal is required for the excessive fork resection observed in ZKSCAN3-deficient cells, we performed DNA fiber experiments in HeLa cells treated with siRNAs targeting ZKSCAN3, SMARCAL1, or both. As shown in Figure 2*A*, fork resection in ZKSCAN3-depleted cells was completely rescued by SMARCAL1 knockdown. SMARCAL1 depletion also rescued the fork resection phenotype in ZKSCAN3-KO cells (Fig. 2*B*). These data support the idea that ZKSCAN3 prevents aberrant processing of reversed replication forks upon replication stress.

To determine which nucleases are involved in fork resection in ZKSCAN3-depleted cells, we performed DNA fiber analysis to examine the effects of functional disruption of Exo1 or Mre11 on fork resection after replication stress. As shown in Figure 2, *C* and *D*, both siRNA-mediated knockdown of Exo1 and Mirin-mediated Mre11 inhibition completely rescued the fork resection phenotype in ZKSCAN3-knockdown or ZKSCAN3-KO cells after HU treatment. These observations suggest that ZKSCAN3 suppresses aberrant fork resection by Exo1 and Mre11 after replication stress. This is similar to BRCA1, which also inhibits excessive fork resection by Exo1 and Mre11 upon replication stress (15).

### The SCAN domain of ZKSCAN3 mediates its role in fork protection under replication stress

**Figure 3:**
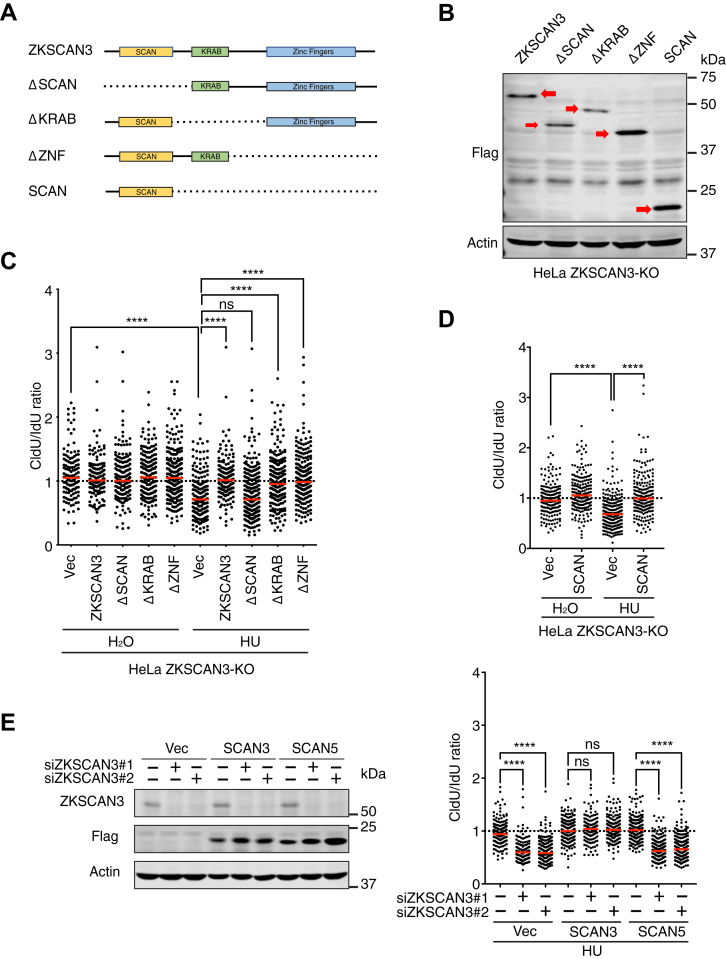
**The SCAN box of ZKSCAN3 mediates its function in fork protection.***A*, schematic diagram of full length and truncation mutants of ZKSCAN3. *B*, expression of N-terminally FLAG-tagged full length and truncation mutants of ZKSCAN3 proteins as depicted in (*A*). *Red arrows* mark the expressed proteins. *C*, fork resection in ZKSCAN3-KO HeLa cells complemented with full length or truncation mutants of ZKSCAN3 after HU treatment (4 mM, 2 h). *Red bars* represent the median. At least 200 tracks were scored for each sample. n = 3, ∗∗∗*p* ≤ 0.0001, ns, not significant (one-Way ANOVA). *D*, fork resection in ZKSCAN3-KO HeLa cells complemented with the SCAN box of ZKSCAN3 after HU treatment (4 mM, 2 h). *Red bars* represent the median. At least 200 tracks were scored for each sample. n = 3, ∗∗∗∗*p* ≤ 0.0001 (one-Way ANOVA). *E*, *Left panel*: Expression of the SCAN box of ZKSCAN3 (SCAN3) or the SCAN box of ZKSCAN5 (SCAN5) in ZKSCAN3-knockdown HeLa cells. *Right panel*: Fork resection in ZKSCAN3-knockdown cells complemented with SCAN3 or SCAN5 after HU treatment (4 mM, 2 h). *Red bars* represent the median. At least 200 tracks were scored for each sample. n = 3, ∗∗∗∗*p* ≤ 0.0001, ns, not significant (one-way ANOVA). HU, hydroxyurea.

To further characterize the function of ZKSCAN3 in fork protection, we next determined which domain(s) in ZKSCAN3 protein mediate its function. Like other ZKSCAN family members, ZKSCAN3 contains three major domains, including SCAN box, KRAB, and ZNF motifs (25). The SCAN box is believed to mediate protein homo/hetero-dimerization, whereas the KRAB and ZNF mediate transcriptional repression and DNA-binding activities, respectively (25). To determine the roles of these domains in fork protection, we expressed N-terminally FLAG-tagged WT and truncation mutants of ZKSCAN3 in ZKSCAN3-KO HeLa cells (Fig. 3, *A* and *B*). DNA fiber analysis was then performed in those replacement cells after HU treatment. In contrast to WT protein, which fully rescued the fork resection phenotype of ZKSCN3-KO cells, a ZKSCAN3 mutant lacking the SCAN box failed to do so, indicating that the SCAN box is important for the function of ZKSCAN3 in fork protection (Fig. 3*C*). Interestingly, cells expressing mutants lacking KRAB or ZNF domains exhibited little or no fork resection after HU treatment, indicating that these two domains are dispensable for fork protection (Fig. 3*C*). Remarkably, expression of the SCAN box alone in ZKSCAN3-KO cells fully rescued the fork resection phenotype (Fig. 3*D*). These results indicate that the SCAN box of ZKSCAN3 is both necessary and sufficient for its function in fork protection. The observation that the KRAB and ZNF domains are not required for fork protection strongly suggests that ZKSCAN3 safeguards replication fork structure independently of its function in transcriptional regulation.

To demonstrate the specificity of the function of the SCAN box of ZKSCAN3 (hereafter referred to as SCAN3) in fork protection, we tested whether the SCAN box of a different family member ZKSCAN5 (hereafter referred to as SCAN5) could also support fork protection in ZKSCAN3-depleted cells upon replication stress. As shown in Figure 3*E*, SCAN3 expression fully rescued the fork resection phenotype of ZKSCAN3-knockdown cells, consistent with the results described above in ZKSCAN3-KO cells. In contrast, a similar level of SCAN5 expression failed to rescue fork resection in ZKSCAN3-depleted cells, demonstrating a unique role of the SCAN box of ZKSCAN3 in fork protection (Fig. 3*E*).

### ZKSCAN3, paradoxically, facilitates excessive fork resection in BRCA1-deficient cells

**Figure 4:**
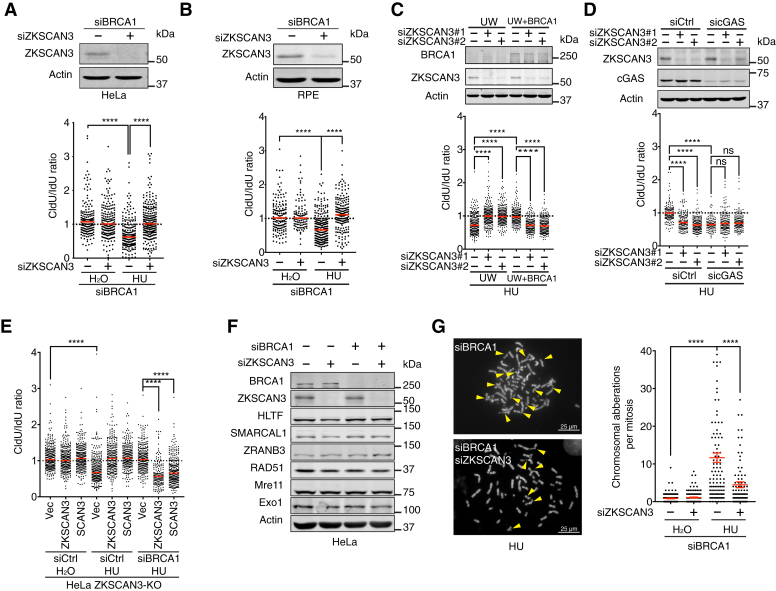
**ZKSCAN3 facilitates fork resection in BRCA1-deficient cells under replication stress.***A*, *Upper panel*: siRNA-mediated knockdown of ZKSCAN3 in BRCA1-depleted HeLa cells. *Lower panel*: Effects of ZKSCAN3 depletion on fork resection in BRCA1-depleted cells after HU treatment (4 mM, 2 h). DNA fiber results were quantified from three independent experiments. *Red bars* represent the median. At least 200 tracks were scored for each sample. ∗∗∗∗*p* ≤ 0.0001 (one-way ANOVA). *B*, *Upper panel*: siRNA-mediated knockdown of ZKSCAN3 in BRCA1-depleted RPE cells. *Lower panel*: Effects of ZKSCAN3 knockdown on fork resection in BRCA1-depleted RPE cells after HU treatment (4 mM, 2 h). DNA fiber results were quantified from three independent experiments. *Red bars* represent the median. At least 200 tracks were scored for each sample. ∗∗∗∗*p* ≤ 0.0001 (one-way ANOVA). *C*, *Upper panel*: siRNA-mediated knockdown of ZKSCAN3 in UW and UW+BRCA1 cells. *Lower panel*: Effects of ZKSCAN3 knockdown on fork resection in UW and UW+BRCA1 cells after HU treatment (4 mM, 2 h). DNA fiber results were quantified from three independent experiments. *Red bars* represent the median. At least 200 tracks were scored for each sample. ∗∗∗∗*p* ≤ 0.0001 (one-way ANOVA). *D*, *Upper panel*: siRNA-mediated knockdown of cGAS in ZKSCAN3-depleted or ZKSCAN3-undepleted HeLa cells. *Lower panel*: Effects of cGAS knockdown on fork resection in ZKSCAN3-depleted HeLa cells after HU treatment (4 mM, 2 h). DNA fiber results were quantified from two independent experiments. *Red bars* represent the median. At least 150 tracks were scored for each sample. ∗∗∗∗*p* ≤ 0.0001, ns, not significant (one-way ANOVA). *E*, effects of SCAN box expression on fork resection in ZKSCAN3-KO HeLa cells depleted of BRCA1 after HU treatment (4 mM, 2 h). DNA fiber results were quantified from three independent experiments. *Red bars* represent the median. At least 200 tracks were scored for each sample. ∗∗∗∗*p* ≤ 0.0001 (one-way ANOVA). *F*, protein levels of HTLF, SMARCAL1, ZRANB3, RAD51, Mre11, and Exo1 in HeLa cells depleted of ZKSCAN3, BRCA1, or both. *G*, effects of ZKSCAN3 knockdown on chromosomal integrity of BRCA1-depleted cells after HU treatment. *Left panel*: Representative images of metaphase chromosome spreads in HU-treated HeLa cells depleted of BRCA1 alone (*upper image*) or depleted of both BRCA1 and ZKSCAN3 (*lower image*). Chromosomal aberrations are marked by *arrows*. *Right panel*: Quantified results of chromosome aberrations in cells depleted of BRCA1 or depleted of both BRCA1 and ZKSCAN3 after HU treatment (4 mM, 6 h). 150 metaphases were examined for each sample. n = 3, ∗∗∗∗*p* ≤ 0.0001 (one-way ANOVA). HU, hydroxyurea.

The fork protection function of ZKSCAN3 described above is similar to that of BRCA1, which also suppresses Exo1/Mre11-dependent aberrant fork resection under replication stress in a SMARCAL1-dependent manner (15). To examine whether ZKSCAN3 acts in the same pathway or in parallel with BRCA1 in fork protection after replication stress, we depleted ZKSCAN3 in BRCA1-knockdown HeLa cells and performed DNA fiber experiments after HU treatment. Consistent with published results, BRCA1-knockdown cells exhibited excessive fork resection after replication stress (Fig. 4*A*). Strikingly, cells depleted of both ZKSCAN3 and BRCA1 exhibited little or no fork resection after HU treatment, compared to cells depleted of BRCA1 alone (Fig. 4*A*). Similar results were obtained in RPE cells, indicating that this unique relationship between ZKSCAN3 and BRCA1 in fork resection regulation is not cell line specific (Fig. 4*B*). To further confirm this finding, we used a BRCA1-deficient cell line UWB 1.289 (hereafter referred to as UW for simplicity) and a reconstituted cell line with BRCA1 re-expression (hereafter referred to as UW+BRCA1) (46). As expected, UW cells exhibited fork resection phenotype after replication stress, which could be rescued by BRCA1 re-expression (see result for UW+BRCA1 cells) (Fig. 4*C*). Consistent with the results described above of BRCA1-knockdown cells, siRNA-mediated knockdown of ZKSCAN3 in UW cells also reversed their fork resection phenotype (Fig. 4*C*). By contrast, ZKSCAN3 depletion resulted in excessive fork resection in UW+BRCA1 cells (Fig. 4*C*). These data strongly suggest that ZKSCAN3 promotes (instead of suppressing) excessive fork resection in BRCA1-deficient cells and that BRCA1 promotes aberrant fork resection in ZKSCAN3-deficient cells. Like BRCA1, the cytosolic DNA sensor protein cGAS has also been shown to suppress aberrant fork resection in the presence of replication stress (47). However, unlike BRCA1-deficient cells, the fork resection phenotype of cGAS-deficient cells could not be reversed by ZKSCAN3 depletion (Fig. 4*D*). This result further demonstrates the unique functional relationship between ZKSCAN3 and BRCA1.

We next asked whether the function of ZKSCAN3 in fork resection regulation in BRCA1-deficient cells is also mediated by its SCAN box. Indeed, expression of SCAN3 completely restored fork resection in cells depleted of both ZKSCAN3 and BRCA1 (Fig. 4*E*). This result again suggests that the role of ZKSCAN3 in fork resection regulation is not mediated through its transcriptional function. In line with this idea, no obvious changes in the protein levels of fork reversal factors (SMARCAL1, HLTF, ZRANB3) and resection nucleases (Exo1 and Mre11) were observed in ZKSCAN3/BRCA1 double-depleted cells compared to single-depleted cells (Fig. 4*F*), suggesting that the rescue effect of ZKSCAN3 depletion on fork resection in BRCA1-deficient cells was not a result of decreased expression of those factors. Consistent with the ability of ZKSCAN3 depletion to rescue fork resection of BRCA1-deficient cells, ZKSCAN3 depletion also partially reversed the chromosomal instability phenotype of BRCA1-knockdown HeLa cells after HU treatment (Fig. 4*G*).

### ZKSCAN3 depletion did not rescue HR defects of BRCA1-deficient cells

**Figure 5:**
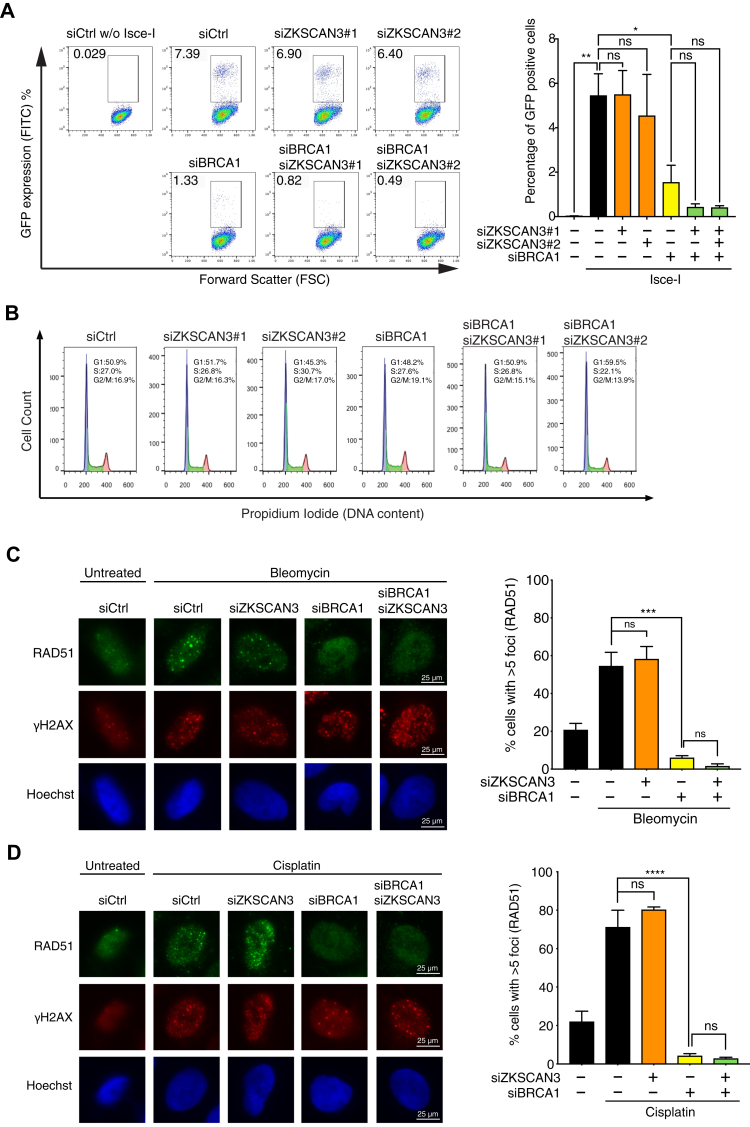
**ZKSCAN3 depletion did not rescue HR defects of BRCA1-deficient cells.***A*, effects of ZKSCAN3 knockdown on HR efficiency in control- or BRCA1-knockdown cells. *Left panel*: U2OS DR-GFP reporter cells were transfected with siRNAs targeting BRCA1, ZKSCAN3, or both, followed by infection with adenoviruses expressing I-SceI. GFP-positive cells were detected through flow cytometry. *Right panel*: Quantified results of the samples depicted in the *left panel*. Data represent mean ± S.E.M. n = 2, ∗*p* ≤ 0.05; ∗∗*p* ≤ 0.01, ns, not significant (one-way ANOVA). *B*, cell cycle profile of U2OS DR-GFP cells transfected with siRNAs targeting BRCA1, ZKSCAN3, or both. *C* and *D*, RAD51 foci in U2OS cells depleted of BRCA1, ZKSCAN3, or both after treatment with bleomycin (12.5 μg/ml, 6 h) (*C*) or cisplatin (10 μM, 6 h) (*D*). *Left panel*: representative images of RAD51 and γH2AX foci after bleomycin or cisplatin treatment. *Right panel*: Percentage of cells with more than five RAD51 foci in samples depicted in the *left panel*. Data represent mean ± S.E.M. n = 3, ∗∗∗*p* ≤ 0.001; ∗∗∗∗*p* ≤ 0.0001; ns, not significant (one-way ANOVA). HR, homologous recombination.

In addition to fork protection, BRCA1 also promotes HR-mediated repair of DSBs, which can be caused by radiation or fork collapse (11, 48). To determine whether ZKSCAN3 depletion can also rescue the HR defects of BRCA1-deficient cells, we used a DR-GFP reporter to measure the efficiency of HR in cells depleted of these proteins (49). A U2OS cell line with an integrated DR-GFP reporter were infected with adenoviruses expressing the endonuclease I-SceI to induce a single DSB within the reporter. Repair of this DSB by HR leads to production of a functional GFP protein in cells, which can be detected by flow cytometry (49). As shown before, siRNA-mediated BRCA1 knockdown drastically reduced HR efficiency (Fig. 5*A*). ZKSCAN3 knockdown alone apparently did not affect HR efficiency (Fig. 5*A*). Cells depleted of both ZKSCAN3 and BRCA1 exhibited a similar level of HR to cells depleted of BRCA1 alone (Fig. 5*A*), suggesting that ZKSCAN3 depletion could not rescue the HR defects caused by BRCA1 loss. Under our experimental conditions, knockdown of BRCA1 and ZKSCAN3 individually or in combination did not cause overt changes in the cell cycle profile, indicating that the observed HR efficiencies in different samples did not result from cell cycle alterations (Fig. 5*B*). To further test whether ZKSCAN3 plays a role in BRCA1-mediated HR, we treated cells with DNA damaging agents bleomycin or cisplatin to generate DSBs and examined the formation of Rad51 foci, which is required for HR (50). ZKSCAN3 depletion did not cause obvious effects on Rad51 foci formation in cells in the presence or in the absence of BRCA1 (Fig. 5*C*), further supporting the idea that ZKSCAN3 is not involved in BRCA1-mediated HR. The inability of ZKSCAN3 depletion to restore the HR in BRCA1-deficient cells may, in part, explain the partial effect of ZKSCAN3 depletion in alleviating chromosomal instability in BRCA1-deficient cells induced by HU (Fig. 4*G*).

### The context-dependent role of ZKSCAN3 in fork resection regulation does not correlate with cellular sensitivity to genotoxic agents

**Figure 6:**
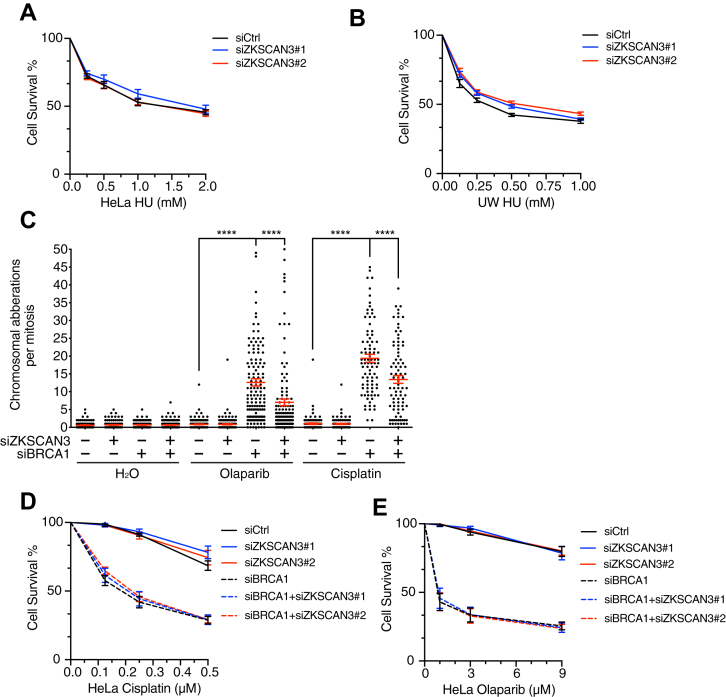
**ZKSCAN3 depletion did not affect the sensitivity of BRCA1-proficient or BRCA1-deficient cells to HU, cisplatin, or Olaparib.***A*, effects of ZKSCAN3 knockdown on the viability of HeLa cells after treatment with indicated doses of HU for 24 h. n = 3, data represent mean ± S.E.M. *B*, effects of ZKSCAN3 knockdown on the viability of BRCA1-deficient UW cells after treatment with indicated doses of HU for 24 h. n = 3, data represent mean ± S.E.M. *C*, result of metaphase chromosome spreading in HeLa cells depleted of ZKSCAN3, BRCA1, or both after treatment with Olaparib (9 μM, 6 h) or cisplatin (0.5 μM, 6 h). One hundred fifty metaphases were examined for each sample. n = 3, ∗∗∗∗*p* ≤ 0.0001 (one-way ANOVA). *D* and *E* effects of knockdown of BRCA1, ZKSCAN3, or both on the viability of HeLa cells after treatment with the indicated doses of cisplatin (*D*) or Olaparib (*E*) for 24 h. n = 3, data represent mean ± S.E.M. HU, hydroxyurea.

Aberrant fork processing and resulting chromosomal instability may cause reduced cell viability in the presence of replication stress. To determine whether the context-dependent function of ZKSCAN3 in fork resection correlates with cell survival, we examined the effects of ZKSCAN3 depletion on the viability of BRCA1-proficient or BRCA1-deficient cells after treatment with HU. As shown in Figure 6*A*, siRNA-mediated ZKSCAN3 depletion did not cause obvious sensitivity to HU in HeLa cells, despite its role in fork protection and chromosomal stability maintenance in the presence of BRCA1. ZKSCAN3 depletion also caused little or no sensitivity of BRCA1-deficient UW cells to HU, although fork protection was restored in those cells (Figs. 6*B* and 4*C*). These results suggest that excessive fork resection and resulting chromosomal aberrations do not always correlate with cellular sensitivity to replication stress.

We also examined the effects of ZKSCAN3 depletion on the sensitivity of BRCA1-deficient cells to Olaparib and cisplatin, both of which are used to treat BRCA1-deficient cancers. Consistent with published results, BRCA1-depleted cells exhibited severe chromosomal instability after Olaparib or cisplatin treatment and were highly sensitive to these chemotherapeutics (Fig. 6, *C*–*E*) (51, 52, 53). ZKSCAN3 depletion partially rescued chromosomal aberrations in BRCA1-depleted cells (Fig. 6*C*). However, ZKSCAN3 depletion did not alter the sensitivity of those cells to cisplatin or Olaparib (Fig. 6, *D* and *E*). These data suggest that restoration of chromosomal stability is not sufficient to cause chemoresistance of BRCA1-deficient cells.

## Discussion

Our study has revealed a context-dependent role of both ZKSCAN3 and BRCA1 in regulating fork processing in the replication stress response. In WT cells, both ZKSCAN3 and BRCA1 function to prevent excessive, Exo1/Mre11-mediated fork resection after replication stress to preserve chromosomal stability (Figs. 1 and 2). However, ZKSCAN3 facilitates fork resection in BRCA1-deficient cells and BRCA1 facilitates fork resection in ZKSCAN3-deficient cells upon replication stress (Fig. 4). These observations highlight a unique relationship between BRCA1 and ZKSCAN3 in fork resection regulation. The functions of ZKSCAN3 in fork resection regulation are mediated by its N-terminal SCAN box and are independent of its role in transcriptional repression (Figs. 3 and 4). Despite its requirement for fork protection and chromosomal stability maintenance, disruption of ZKSCAN3 function does not impact short-term survival of BRCA1-proficient cells in the presence of replication stress (Figs. 1 and 6). Likewise, although ZKSCAN3 depletion rescues the fork resection phenotype of BRCA1-deficient cells, it does not rescue their sensitivity to chemotherapeutics such as Olaparib and cisplatin (Figs. 4 and 6). These findings suggest that aberrant fork resection and the resulting chromosomal instability do not always lead to reduced cell viability and that restoration of fork protection does not necessarily give rise to chemoresistance of BRCA1-deficient cancers.

The identification of novel functions of ZKSCAN3 in fork processing and genome maintenance has expanded its physiological functions and shed new light on its cancer relevance. ZKSCAN3 has been shown to regulate autophagy, lysosome biogenesis, and heterochromatin maintenance (31, 32). Our study indicates that ZKSCAN3 also acts to safeguard the genome (in BRCA1-proficient cells) by suppressing aberrant replication fork processing (Fig. 1). Consistent with the notion that excessive fork resection upon replication stress is dependent on fork reversal (45), we found that depletion of SMARCAL1 rescued the fork resection phenotype of ZKSCAN3-deficient cells (Fig. 2, *A* and *B*). Like BRCA1, ZKSCAN3 acts to prevent aberrant fork resection by Exo1 and Mre11 (Fig. 2, *C* and *D*) (15). ZKSCAN3 functional domains have not been characterized before, but the KRAB domain is likely important for its functions in regulating autophagy, lysosome biogenesis, and heterochromatin maintenance because those functions require ZKSCAN3’s transcriptional function and/or its interaction with KAP1, both of which likely involve the KRAB (31, 32). Surprisingly, the function of ZKSCAN3 in fork protection is mediated solely by its N-terminal SCAN box and does not require the KRAB and ZNF domains (Fig. 3), indicating that the role of ZKSCAN3 in fork resection is mechanistically separable from its other functions. As a protein dimerization domain, SCAN may facilitate fork protection *via* homodimerization or interaction with another SCAN-containing protein (27, 28, 29, 30). However, it is also possible that the SCAN box in ZKSCAN3 mediates interactions with proteins without SCAN that are important for fork protection. We have not observed the association of ZKSCAN3 with chromatin before or after replication stress, raising the possibility that it does not act at the forks to prevent aberrant fork processing. In addition to ZKSCAN3, our siRNA-mediated screen also identified ZKSCAN4 as a potential fork protection factor (Fig. 1*B*). Little is known about the functions of ZKSCAN4, but it shares highest similarity with ZKSCAN3 in protein sequence among the 25 ZKSCAN genes in human cells (54). However, these two proteins apparently do not function redundantly in fork protection, because siRNA-mediated ZKSCAN4 knockdown caused fork resection phenotype without affect the protein levels of ZKSCAN3 (**data not shown**). Future work is needed to further define the roles of ZKSCAN3 and ZKSCAN4 and their functional relationship in replication fork protection.

Our study has also provided additional evidence for the emerging concept that excessive fork resection after replication stress does not necessarily impact the chemosensitivity of BRCA1-proficient or BRCA1-deficient cells (Fig. 6). Despite the critical importance of ZKSCAN3 in fork protection and chromosomal stability, no overt sensitivity of ZKSCAN3-depleted cells to HU-induced replication stress was observed (Fig. 6*A*). A similar phenotype was observed for BRCA2, whose depletion causes aberrant fork resection but little sensitivity to HU (55). By contrast, depletion of a number of other fork protection factors such as BRCA1, RIF1, AMPK, BOD1L led to heightened sensitivity to HU (7, 9, 14, 56). These observations suggest that the impact of fork resection on cell survival in the presence of replication stress is dependent on specific genetic contexts. Recent studies suggest that reversal of the fork resection phenotype in BRCA2-deficient cells can result in resistance to PARPi (20, 57, 58, 59, 60). In support of this idea, functional disruption of PTIP, CHD4, or EZH2 reversed both fork resection and the PARPi sensitivity of BRCA2-deficient cells (20, 61, 62). However, in BRCA1-deficient cells, silencing RADX, EZH2, or MUS81 prevented excessive fork resection after replication stress without reversing the PARPi sensitivity of those cells (15, 62, 63). Similarly, our results indicate that ZKSCAN3 depletion also rescued the chromosomal instability phenotype of BRCA1-deficient cells without affecting their sensitivity to PARPi or cisplatin (Figs. 4 and 6). These observations suggest a context-dependent role of fork resection and chromosomal instability in the modulation of the chemoresistance of BRCA-deficient cancer. The chromosomal instability caused by ZKSCAN3 deficiency may have long-term impact on cancer formation and progression. Further dissection of the mechanisms and regulation of fork protection will advance our understanding of genome maintenance and the development of new cancer treatment strategies.

## Experimental procedures

### Cell culture

HeLa, U2OS, and HEK293T cells were cultured in Dulbecco's Modified Eagle Medium (DMEM) with 10% fetal bovine serum, 100 U/ml penicillin, and 100 μg/ml streptomycin in a humidified incubator containing 5% CO2 at 37 °C. DMEM/Nutrient Mixture F-12 medium (DMEM/F-12) was used for culturing nontransformed RPE cells, as previously described (7). UWB 1.289 cells and UWB 1.289 cells reconstituted with BRCA1 (gifts of Dr Alessandro Vindigni) were cultured at 37 °C with 5% CO2 in 50% RPMI media, 50% Mammary Epithelial Cell Growth Medium BulletKit (Lonza CC-3150) supplemented with 3% fetal bovine serum, 100 U/ml penicillin, and 100 μg/ml streptomycin.

### Generation of ZKSCAN3 mutants and ZKSCAN3-KO and ZKSCAN3-expressing cells

Flag-tagged human full-length ZKSCAN3 and its truncation mutants were inserted into the pCDH vector *via* PCR and the Gibson Assembly cloning methods. siRNA-resistant ZKSCAN3 construct (with three point mutations) was made by site-directed mutagenesis. To generate HeLa ZKSCAN3-KO cell line, a construct expressing sgRNA (5′ CACCGCACTATTGACACCAGCCCCA 3′) in pLenti-CRSIPRv2 (Addgene #52961) was transfected into HeLa cells *via* Lipofectamine 3000 (Life Technologies). Twenty four hours after transfection, cells were selected by puromycin (2 μg/ml) for 48 h. Single cells were seeded and grown in 96-well plates for amplification. Knockout single clones were verified by Western blot. HeLa ZKSCAN3-KO cells stably expressing full length ZKSCAN3 or its truncation mutants were generated through lentiviral infection. Lentiviral vectors expressing full length ZKSCAN3 or truncation mutants were contransfected into HEK293T cells together with pPAX2 and pVSVG plasmids. Filtered lentivirus-containing medium was used to infect HeLa ZKSCAN3-KO cells. Seventy two hours after infection, the cells were selected by puromycin (2 μg/ml) for 48 h. Expression of ZKSCAN3 proteins were verified by Western blot using an anti-FLAG or anti-ZKSCAN3 antibody.

### siRNA-mediated knockdown of gene expression

siRNA was transfected using lipofectamine RNAiMAX (Thermo Fisher Scientific) according to the manufacturer’s instructions. siRNAs used in this study include BRCA1 (Thermo Fisher, s459), Exo1 (Thermo Fisher, s17502), SMARCAL1 (Thermo Fisher, s26996), ZKSCAN3#1 (Thermo Fisher, s37205), ZKSCAN3#2 (Thermo Fisher, s37206), cGAS (Thermo Fisher, s41746), and Control (negative control, Thermo Fisher, 12935146). siRNAs used for the ZKSCAN family screen are listed in Table 1.

**Table 1:** (Related to Experimental procedures)

siRNAs	Source
siRNA for ZKSCAN1 #1	Thermo Fisher, siRNA ID: s15081
siRNA for ZKSCAN1 #2	Thermo Fisher, siRNA ID: s15082
siRNA for ZKSCAN2 #1	Thermo Fisher, siRNA ID: s50897
siRNA for ZKSCAN2 #2	Thermo Fisher, siRNA ID: s50898
siRNA for ZKSCAN3 #1	Thermo Fisher, siRNA ID: s37205
siRNA for ZKSCAN3 #2	Thermo Fisher, siRNA ID: s37206
siRNA for ZKSCAN4 #1	Thermo Fisher, siRNA ID: s51828
siRNA for ZKSCAN4 #2	Thermo Fisher, siRNA ID: s51829
siRNA for ZKSCAN5 #1	Thermo Fisher, siRNA ID: s24298
siRNA for ZKSCAN5 #2	Thermo Fisher, siRNA ID: s24300
siRNA for ZKSCAN6 #1	Thermo Fisher, siRNA ID: s15039
siRNA for ZKSCAN6 #2	Thermo Fisher, siRNA ID: s15040
siRNA for ZKSCAN7 #1	Thermo Fisher, siRNA ID: s31718
siRNA for ZKSCAN7 #2	Thermo Fisher, siRNA ID: s31719
siRNA for ZKSCAN8 #1	Thermo Fisher, siRNA ID: s15257
siRNA for ZKSCAN8 #2	Thermo Fisher, siRNA ID: s15258
siRNA for ZKSCAN9 #1	Thermo Fisher, siRNA ID: s19814
siRNA for ZKSCAN9 #2	Thermo Fisher, siRNA ID: s19815
siRNA for ZKSCAN10 #1	Thermo Fisher, siRNA ID: s15272
siRNA for ZKSCAN10 #2	Thermo Fisher, siRNA ID: s15273
siRNA for ZKSCAN11 #1	Thermo Fisher, siRNA ID: s15290
siRNA for ZKSCAN11 #2	Thermo Fisher, siRNA ID: s15291
siRNA for ZKSCAN12 #1	Thermo Fisher, siRNA ID: s19706
siRNA for ZKSCAN12 #2	Thermo Fisher, siRNA ID: s19707
siRNA for ZKSCAN13 #1	Thermo Fisher, siRNA ID: s32956
siRNA for ZKSCAN13 #2	Thermo Fisher, siRNA ID: s32957
siRNA for ZKSCAN14 #1	Thermo Fisher, siRNA ID: s38521
siRNA for ZKSCAN14 #2	Thermo Fisher, siRNA ID: s38522
siRNA for ZKSCAN15 #1	Thermo Fisher, siRNA ID: s51456
siRNA for ZKSCAN15 #2	Thermo Fisher, siRNA ID: s51457
siRNA for ZKSCAN16 #1	Thermo Fisher, siRNA ID: s46070
siRNA for ZKSCAN16 #2	Thermo Fisher, siRNA ID: s46071
siRNA for ZKSCAN17 #1	Thermo Fisher, siRNA ID: s39497
siRNA for ZKSCAN17 #2	Thermo Fisher, siRNA ID: s39498
siRNA for ZKSCAN18 #1	Thermo Fisher, siRNA ID: s25008
siRNA for ZKSCAN18 #2	Thermo Fisher, siRNA ID: s25009
siRNA for ZKSCAN19 #1	Thermo Fisher, siRNA ID: s21180
siRNA for ZKSCAN19 #2	Thermo Fisher, siRNA ID: s21181
siRNA for ZKSCAN20 #1	Thermo Fisher, siRNA ID: s31200
siRNA for ZKSCAN20 #2	Thermo Fisher, siRNA ID: s31201
siRNA for ZKSCAN21 #1	Thermo Fisher, siRNA ID: s15284
siRNA for ZKSCAN21 #2	Thermo Fisher, siRNA ID: s15285
siRNA for ZKSCAN22 #1	Thermo Fisher, siRNA ID: s10291
siRNA for ZKSCAN22 #2	Thermo Fisher, siRNA ID: s10292
siRNA for ZKSCAN23A #1	Thermo Fisher, siRNA ID: s50566
siRNA for ZKSCAN23A #2	Thermo Fisher, siRNA ID: s50567
siRNA for ZKSCAN23B #1	Thermo Fisher, siRNA ID: s35242
siRNA for ZKSCAN23B #2	Thermo Fisher, siRNA ID: s35243
siRNA for ZKSCAN24 #1	Thermo Fisher, siRNA ID: s15117
siRNA for ZKSCAN24 #2	Thermo Fisher, siRNA ID: s15118

### Nondenaturing BrdU immunofluorescence staining for measuring fork resection

HeLa cells were cultured on glass-bottomed dishes then incubated with BrdU (10 μM) for 36 h before treatment with HU (2 mM) for 5 h to induce replication stress. Cells were then permeabilized with extraction buffer (10 mM Pipes pH 6.8, 100 mM NaCl, 300 mM sucrose, 3 mM MgCl2, 1 mM EGTA, and 0.2% Triton X-100) for 5 min. After this, cells were incubated sequentially with 3% paraformaldehyde in phosphate-buffered saline (PBS) (room temperature) for 20 min, cold methanol (- 20 °C) for 20 min, and then ice-cold acetone (4 °C) for 30 s. Subsequently, cells were incubated with blocking buffer (PBS containing 0.05% Tween-20 and 2% bovine serum albumin (BSA)) for 1 h at room temperature, followed by incubation with anti-BrdU antibody (1:1000, BD Pharmingen, 555627) overnight at 4 °C. Cells were then incubated with goat anti-mouse secondary antibody (1:500, Invitrogen, A11001) for 1 h at room temperature. Both primary and secondary antibodies were diluted in PBS containing 2% BSA. After staining with Hoechst 33342 (1 μg/ml), images were captured using an inverted microscope (Nikon Ti-E) with a 20× objective and Metamorph software (Molecular Devices). The BrdU signal in individual nuclei (defined by Hoechst-stained area) was determined using ImageJ. Cells with a BrdU signal above that in the majority (98%) of HU-untreated control cells were taken as BrdU-positive. For each sample, 1000 randomly selected cells were analyzed. Statistical analysis was performed in GraphPad Prism 9.0 using an unpaired *t* test.

### DNA fiber assay for measuring fork resection

HeLa, RPE, or UW cells were pulse-labeled sequentially with thymidine analogs IdU (20 mM) for 20 min and then with CldU (200 mM) for 20 min. Cells were then washed with PBS and treated with HU (4 mM) for 2 h to induce replication stress. After this, cells were trypsinized and resuspended in ice-cold PBS at 500,000 cells/ml (for HeLa cells) or 1,000,000 cells/ml (for RPE and UW cells). Two microliters of cell suspension were spotted onto a precleaned glass slide and lysed with eight μl of spreading buffer (0.5% SDS in 200 mM Tris-HCl, pH 7.4, and 50 mM EDTA). After 6 min of incubation, the slides were tilted to spread the genomic DNA. Slides were air-dried and then fixed in precold methanol and acetic acid (3:1) for 10 min and stored overnight. The next day, slides were rehydrated in water for 5 min and denatured with 2.5 M HCl for 1h at room temperature. Slides were then rinsed in PBS, blocked in 5% BSA with 0.1% Tween-20 in PBS for 1h at room temperature, and incubated with rat anti-BrdU (1:500, Novus or Abcam) and mouse anti-BrdU (1:50, Becton Dickinson) in a humid chamber for 2 h at room temperature. After incubation, slides were washed in PBS with 0.1% Tween-20 and stained with Alexa Fluor 488-labeled goat anti-mouse antibody and Alexa Fluor 594-labeled goat anti-rat antibody (1:100 each, Thermo Fisher Scientific). Slides were finally mounted in Prolong Gold Antifade (Thermo Fisher Scientific). Replication tracks were imaged with a 60× objective fluorescence microscope (inverted Nikon Ti-E microscope) and measured using ImageJ software. At least 150 to 200 individual tracks were measured in each sample, and results were analyzed in Graphpad Prism 9.0.

### Metaphase chromosome spreading assay

Cells were seeded in six-well plates, treated with 4 mM HU (Sigma-Aldrich), 0.5 μM cisplatin (Sigma-Aldrich), or 9 μM Olaparib (SelleckChem) for 6 h and then recovered in fresh medium for 20 h before being treated with nocodazole (0.2 mg/ml) for 4 h to induce cell cycle arrest. Cells were then trypsinized and harvested and then incubated in 75 mM KCl in PBS for 10 min at 37 °C followed by fixation in a methanol/acetic acid (3:1) solution. After overnight incubation at 4 °C, the fixed cell suspension was dropped onto slides to obtain chromosome spreads, which were then stained with Giemsa (Sigma-Aldrich). Images of metaphase spreads were captured through microscopy using a 60× objective. For each condition, at least 50 metaphases were examined, and results were analyzed in Graphpad Prism 9.0.

### HR assay

U2OS cells stably expressing a DR-GFP HR reporter were transfected with a control siRNA or siRNAs targeting BRCA1, ZKSCAN3, or both. Two days after transfection, cells were infected with adenovirus expressing I-SceI nuclease to introduce a single DSB within the reporter. Three days after infection, cells were trypsinized and washed with PBS, and the GFP signal in live cells was measured using BD FACS Calibur Flow Cytometer and analyzed using FlowJo. To analyze the cell cycle, the remaining cells from GFP analysis were fixed with 70% ethanol for 30 min on ice, washed with cold PBS, and then incubated with PBS containing propidium iodide (20 μg/ml) and RNase A (200 μg/ml) at 37 °C for 30 min. Flow cytometry was then performed using BD FACS Calibur Flow Cytometer and cell cycle profile was analyzed using FlowJo.

### Alamar Blue assay and CCK8 assay for measuring cell viability

Cells were seeded in 24-well plates at a density of 10,000 cells/well a day after transfection and treated with cisplatin (Sigma-Aldrich) or Olaparib (SelleckChem) at the indicated concentrations for 24 h. Three or four days after treatment, cells were incubated with 1× Alamar Blue staining buffer and then transferred into 96-well plates. Cell number was measured through the absorbance at 595 nm in a microplate reader and Gen5 version 2.09 software (BMG LABTECH). Cell Counting Kit-8 assay (APE x BIO) was performed according to the manufacturer’s protocol. The cell number was determined through the absorbance at 450 nm using the same plate reader and software. Statistical analysis was performed in GraphPad Prism 9.0.

### Immunofluorescence staining and immunoblotting

Immunofluorescence staining was performed as previously described (7). Briefly, cells cultured on glass coverslips were washed with PBS once, then permeabilized with 0.2% Triton X-100 in PBS (PBST) for 10 min at room temperature. After three washes with PBS, cells were fixed with 4% paraformaldehyde for 10 min at room temperature and then blocked with 10% goat serum in PBS for 1 h at room temperature. Coverslips were incubated overnight at 4 °C with primary antibodies diluted in PBS containing 10% goat serum and 0.1% Tween-20. Coverslips were then washed with PBST three times, then incubated at room temperature for 1 h in the dark with secondary antibodies diluted in PBS containing 10% goat serum and 0.1% Tween-20. After three washes in PBST, cells were stained with Hoechst (1 mg/ml) for 10 min at room temperature before mounting with Prolong gold mounting solution. Fluorescence images were captured using a 60× objective. For each condition, at least 100 cells were quantified in a blind manner, and results were analyzed in Graphpad Prism 9.0.

For immunoblotting, cells were collected and lysed in 1× Loading Buffer with beta-mercaptoethanol. Total proteins were separated by 8–12% SDS-PAGE for 1 h and then transferred onto PVDF membranes for 1 to 2 h depending on proteins’ molecular weights. After blocking with 1× casein buffer for 40 min, the membrane was incubated with primary antibodies overnight at 4 °C and then with secondary antibodies (1:10,000, Thermo Fisher Scientific). Western blot signal was detected using an Odyssey scanning system (Licor). Primary antibodies were used at the following dilutions: anti-ZKSCAN3 (1:100, Santa Cruz Biotechnology), anti-BRCA1 (1:1,000, Abclonal), anti-SMARCAL1 (1:1,000, Cell Signaling Technology), anti-HLTF (1:1,000, Abclonal), anti-ZRANB3 (1:1,000, Abclonal), anti-Exo1 (1:1,000, EMD Millipore), anti-Mre11 (1:1,000, Oncogene Research Products), anti-Rad51 (1:100, Santa Cruz Biotechnology), anti-Flag (1:1,000, Abclonal), anti-cGAS (1:1,000, Cell Signaling Technology), and anti-beta Actin (1:5,000, Cell Signaling Technology). Beta-Actin was used as a loading control.

### Statistical analysis

Statistical analyses were performed using GraphPad Prism 9.0. The tests performed, the sample size (n), and the number of independent replicates for each experiment are described in the figure legends.

## Data availability

All data are contained within the article.

## Conflict of interest

The authors declare no competing financial interests.
